# Bilingual education searching for promising didactic proposals

**DOI:** 10.3389/fpsyg.2014.01359

**Published:** 2014-12-05

**Authors:** Ibon Manterola

**Affiliations:** Department of Linguistics and Basque Studies, Universidad del País Vasco/Euskal Herriko UnibertsitateaVitoria-Gasteiz, Spain

**Keywords:** bilingual education, instructional strategies, integrated didactics of languages, text genres, didactic sequences

In the Basque context, the English-French bilingual education systems from Canada and Quebec are considered an indispensable reference for the Basque-Spanish and Basque-French bilingual schools, which, for the approximately last 40 years, constitute a keystone for the revitalization process of the Basque language (Zalbide, 1998). One of the crucial contributions of English-French bilingual education was to confirm that it was possible to successfully provide schooling in a second language, given some sociolinguistic, didactic, and psycholinguistic conditions. Research on French immersion showed that not only would children successfully develop the L2 or language of instruction, but also the L1 or family language and moreover, positive academic results would be obtained (Genesee, 2006). From the Basque perspective, these findings marked a milestone to support immersion schooling in Basque for Spanish or French L1 children and obviously constituted a very powerful argument in order to defend a similar system for the Basque Country (Idiazabal, 2003). It should be noted that in this new context of bilingual education and more specifically immersion education, the specificity of the Basque case was and still is that one of the languages involved is a minority language. We consider that this is not a minor fact since the Basque case is considered a very interesting example of bilingual and immersion education from the perspective of minority language education (Idiazabal et al., 2008; Cenoz, 2009).

One of the core assumptions for immersion pedagogy was that children should be provided as much contact and input with the immersion language as possible and this implied that the family language should not be present in the classroom or that its use should be minimal, keeping the language of immersion and the family language separate. This is what is called the monolingual instructional approach (Cummins, 2008). However, recent research not only in immersion but also in other bilingual and multilingual approaches proposes going beyond these assumptions and adopting a more bilingual or even multilingual didactic perspective. Cummins claims that new opportunities appear for bilingual instructional strategies in order to promote cross-linguistic transfer in bilingual students. Cummins not only mentions translations, but he also refers to the use of students' L1 in very precise stages in the production of dual language identity texts.

A bilingual or even multilingual didactic perspective is also adopted by the so-called plural approaches to languages and cultures (Troncy, 2014), which supports teaching and learning activities that imply the use of more than one linguistic variety (i.e., more than one language but also more than one variety within a single language). One of the main proposals within this approach is that children need to work with more than one language together and the core type of activity is the comparison across languages in metalinguistic activities (Candelier et al., 2012).

In our opinion, the paper by Naqvi et al. (2014) can be included in this trend toward bilingual and multilingual didactics. By offering a compact synthesis of diverse types of data (video observations, questionnaires, students' responses etc.), it permits to go beyond the “monolingual solitude assumption” in Spanish (L2)-English (L1) bilingual education. Naqvi et al. show that dual language book reading sessions provide many opportunities to work on the similarities and differences between Spanish and English and by doing so, they clearly enhance the development of metalinguistic awareness. Another type of activity studied in the paper refers to video-literacy projects in which children use both L1 and L2 as a strategy to foster cross-linguistic transfer. Overall, a more integrated model of language teaching and learning is proposed in order to exploit cross-linguistic transfer. And this requires several didactic challenges, i.e., new models of task design and strategies to successfully combine L1 and L2 in classroom activities.

Another didactic approach that is very in line with the previous ideas is the integrated didactics of languages. It consists on the design of multilingual curricula and teaching materials, and according to Cavalli (2005), its origins can already be found in 1973, when the Council of Europe claimed that the efforts to establish the links between the teaching of the mother tongue and the teaching of other languages were far from being enough. So language teachers should be able to coordinate their pedagogical activities and base their teaching on common linguistic principles. Roulet (1980) specifies that the coordination between languages covers the curricula and teaching materials, linguistic terminology, and the design and realization of classroom activities.

As it can be appreciated, the coordination and combination between languages is conceived not only in very precise classroom activities but also in a more macro level such as the design of teaching materials and curricula. For instance, in the Andorran multilingual education Catalan is the language of instruction in the first 2 years of nursery school, that is to say, from ages 2 to 4 (Dolz and Wharton, 2008). French is added to Catalan as the language of instruction between ages 4 and 6. Within the first cycle of primary school (ages 6–8) Catalan is used to teach reading, writing, and all the subjects. French is used in some subjects but only orally and for a very initial level of reading and writing. Both Catalan and French are the languages of instruction during the second cycle of primary school (ages 8–10) and English is introduced as a subject. At ages 10 and 12 Spanish is included as the third language of instruction (and also as a subject, together with English). Finally, within the secondary school the four languages are taught as subjects and at least one subject other than a linguistic course is taught in French and in Spanish. Most subjects are taught in Catalan but the aim for the future is to increase the number of subjects taught in other languages.

In our opinion, one of the most interesting contributions of the Andorran case to the field of multilingual didactics is that it is based on didactic sequences and text genres (Dolz and Schneuwly, 1998; Bronckart, 2007)^1^. A didactic sequence is constituted by sequentially organized classroom activities whose main aim is to work on the production and comprehension of a text genre (written fairy tale, public debate, written or oral recipe, letter to the editor, etc.). The work on genres is always included within projects where the communicative context is always very clearly specified. Didactic sequences begin with the presentation of the communicative project (for instance, to create a multicultural recipe book that will be distributed to the school community), and are constituted by the production of initial texts, subsequent sets of activities focusing on difficulties identified in the initial texts, and the production of a final text which permits to assess the progression of the students and the relevance and efficiency of the activities designed by the teacher.

A recent experimental study in the field of integrated didactics of languages carried out in the Basque Country shows that a multilingual didactic sequence carried out mainly in Basque but containing some activities in English and Spanish can be successful for the learning of the three languages (Badiola et al., 2014). This research also shows that transfer plays a central role. Participants were 16-year-old multilingual students whose first language is Spanish and who live in a very predominant Spanish-speaking sociolinguistic context. Basque is their second language and the main language of instruction at school. Finally, English constitutes the third language. The text genre chosen for the didactic sequence was the short biography, which had to be produced in three languages. Within the activities of the multilingual didactic sequence very precise discursive skills were targeted (such as the organization of the contents, the production of text organizers and the reference to characters). The organization of the contents and the reference to the characters were only worked in Basque but in the final productions students not only improved the texts in Basque but also the ones in Spanish and English. Text organizers were worked in each language and overall they showed a general improvement in the final texts in three languages. The authors emphasize that the transfer of discursive skills was from the L2 of students (Basque) to the L1 and L3. We would add that this constitutes a remarkable fact since in this case the L2 is not a language of international presence such as Spanish, English or French but rather a minority language of very limited social use in a revitalization process.

The main goals of bilingual and multilingual education include three fundamental aspects of linguistic education (Idiazabal et al., 2015): the promotion of multilingual competence to use languages in diverse communicative contexts, the development of metalinguistic skills and the development of positive attitudes toward linguistic diversity in general and toward minority languages in particular. As it is argued by Naqvi et al. (2014) it is necessary to go beyond the monolingual assumption in bilingual education and precisely their research shows that bilingual instructional strategies can be successful in promoting bilingual competence and metalinguistic awareness. Along the same line, research carried out in the field of integrated didactics of languages shows that the coordination and combination between languages is also possible at the curriculum level. And it also suggests that the teaching of text genres within bilingual didactic sequences constitutes a promising didactic proposal for bilingual education.

## Conflict of interest statement

The author declares that the research was conducted in the absence of any commercial or financial relationships that could be construed as a potential conflict of interest.

## Footnotes

^1^Another interesting example can be found in the Italian Aosta Valley where Italian and French bilingual didactic sequences were designed (Joly et al., 2004).
